# Herbal Formula Modified Buzhong-Yiqi-Tang for Functional Constipation in Adults: A Meta-Analysis of Randomized Controlled Trials

**DOI:** 10.1155/2018/9602525

**Published:** 2018-01-16

**Authors:** Hanlin Gong, Feng Qin, Hongbo He

**Affiliations:** ^1^Department of Integrated Traditional Chinese and Western Medicine, West China Hospital, Sichuan University, Chengdu 610041, China; ^2^Research Core Facility, West China Hospital, Sichuan University, Chengdu 610041, China

## Abstract

**Background:**

Herbal formula Modified Buzhong-Yiqi-Tang (MBYT) has been widely used for the treatment of functional constipation in East Asia, but its efficacy and safety are unclear.

**Methods:**

The study was to evaluate the efficacy and safety of MBYT for adult patients with functional constipation. Randomized clinical trials were selected according to predefined inclusion and exclusion criteria.

**Results:**

In total, twenty-five randomized controlled clinical trials were included with 2089 patients. There was evidence that MBYT treatment significantly improved the symptoms of functional constipation compared with stimulant laxatives, osmotic laxatives, and prokinetic agents. Our results also demonstrated that, when used as an adjuvant therapy, MBYT significantly improved the symptoms of functional constipation, when compared with osmotic laxatives alone, prokinetic agents alone, and biofeedback alone. Moreover, patients taking MBYT experienced fewer adverse events compared to the control groups.

**Conclusion:**

This review suggests that MBYT appears to have excellent therapeutic effect on adult patients with functional constipation and no serious side effects were identified. However, due to overall limited quality, the therapeutic benefit of MBYT may be substantiated to a limited degree. Better methodological quality and large controlled trials are expected to further quantify the therapeutic effect of MBYT.

## 1. Introduction

Functional constipation is a very common condition that affects a considerable proportion of the population of all ages. The prevalence of the condition ranges between 0.7% and 79%, with an average rate of 16% on a global scale [1]. On the mainland of China, about 6% of population experience functional constipation, of which women and the elderly are at highest risk [2]. The pathogenesis of functional constipation is very complicated, and its pathophysiological mechanism is not yet clear. According to the result of epidemiological studies, it showed that the functional constipation was relevant to lifestyle, eating habits, gender, age, autonomic nervous dysfunction, and other factors [3, 4]. Long-term constipation may induce hemorrhoids and cardiovascular disease, increase the risk of colon cancer, and cause depression and anxiety and other interest exceptions. Thus, functional constipation greatly affects patients' quality of life with increasing medical burden [5, 6].

Modified Buzhong-Yiqi-Tang (MBYT) is a well-known Chinese herbal formula that has long been used by local people for the treatment of gastrointestinal diseases, cancer, and chronic fatigue syndrome associated with the syndrome of “sinking of qi due to spleen deficiency” (the concepts of traditional Chinese medicine) [7–9]. The preparation is developed from Bu-Zhong-Yi-Qi-Tang (called Bo-jung-ik-gi-tang in Korea or called Hochu-ekki-to in Japanese), which is a classic herbal formula, originally recorded in “Piwei Lun” (a medical literature, written in Jin Dynasty, AD 1247). MBYT mainly includes the following eight Chinese herbs: Huangqi [the dried roots of* Astragalus membranaceus* (Fisch) Bge], Baizhu [the dried rhizome of* Atractylodes macrocephala* Koidz (Asteraceae)], Chenpi (the dried mature fruit peels of* Citrus reticulata* and* Citrus sinensis*), Renshen [the dried roots of* Panax ginseng* C.A. Meyer (Araliaceae)], Chaihu (the dried roots of* Bupleurum falcatum* L.), Shengma (the dried rhizome of* Cimicifuga foetida* L.), Zhigancao (the processed dried roots or rhizomes of* Glycyrrhiza uralensis* Fisch.,* G. inflata* Bat., or* G. glabra* L.), and Danggui [the dried roots of* A. sinensis* (Oliv.) Diels] [8, 9]. Additionally, astragaloside IV, calycosin, glycyrrhizic acid, enoxolone, saikosaponin D, ferulic acid, and hesperiden have been identified within the preparation and biological samples, and these components may be used as markers for quality control of MBYT [10].

In experimental studies, MBYT was shown to significantly improve gastrointestinal hormone levels and promote gastrointestinal motility and gastric emptying and regulation of the immune function [11–13]. The foremost merits of traditional herbal formula are its low cost, good compliance, few side effects, and high patient satisfaction in China, when long-term use is involved. Recent many studies have suggested that MBYT and its extracts revealed the beneficial effects for functional constipation. For the acceptance and application of traditional herbal formula, the greatest hindrance in the Western world is the scientific evaluation. Therefore, it is important to make scientific evaluation standard system to MBYT general. Despite the extensive use of MBYT in East Asian countries, most of the evidence about MBYT is anecdotal and has not been properly studied with scientifically rigorous trials. The efficacy and safety of MBYT treatment need to be reviewed to inform clinical practitioner and the areas for new research on MBYT ought to be highlighted.

Therefore, we have recently reviewed available evidence on MBYT to offer guidance for the treatment of functional constipation. The result would be helpful to assess the effectiveness and safety of MBYT on functional constipation.

## 2. Methods

### 2.1. Literature Search

The randomized controlled trials (RCTs) were identified from the PubMed (1966 to Dec 2016), Cochrane Controlled Trials Register (the Cochrane Library 2017, Issue 1), and Embase (1980 to Dec 2016) through Ovid; China National Knowledge Infrastructure (CNKI, 1994 to Dec 2016), Wanfang Data (1989 to Dec 2016), and Chinese Scientific Journals Database (VIP, 1990 to Dec 2016). A search strategy to locate studies on functional constipation was structured as “Buzhongyiqi” or “Buzhong Yiqi” or “Bu Zhong Yi Qi” or “Hochuekkito” or “Hochu-ekkito” or “Hochu-ekki-to” or “TJ 41” or “Bojungikgitang” or “Bojungikgi” or “Bo-jung-ik-gi”. In order to gather the largest number of papers, we included any relevant RCTs, regardless of the language of publication. We scanned bibliographies of relevant studies for possible references to additional clinical trials.

### 2.2. Inclusion Criteria

Studies were included in the meta-analysis if they met the following criteria: (1) study design: all participants were randomly allocated to intervention and control groups, both parallel and crossover studies were eligible; (2) target population: all participants were aged 18 years and older; (3) diagnostic criteria: all participants were diagnosed as having functional constipation according to the Rome I/II/III diagnostic criteria or other diagnostic criteria for functional constipation; (4) comparison: studies had to compare MBYT with other treatment (such as placebo, stimulant laxatives, osmotic laxatives, prokinetic agents, and biofeedback therapy); (5) outcome: studies have used dichotomous data based on total effective rate or symptom scores as primary outcomes.

### 2.3. Exclusion Criteria

Case reports, animal studies, nonclinical outcome studies, and reviews were excluded. Case series or clinical trials regarding the efficiency and safety of MBYT on functional constipation were also excluded if they (1) were studies in children (<18 years) only; (2) included an inappropriate diagnosis standard for functional constipation; (3) were unverified RCT studies; (4) included no appropriate control group, and (5) were duplicate publications.

### 2.4. Study Selection

Two reviewers (H.G. and F.Q.) were involved in data collection and management following a four-step approach, and they were performed independently. Frist, the titles and abstracts of the relevant articles were screened to see if they met the selection criteria. Second, full texts of the relevant articles were reviewed according to the predefined inclusion or exclusion criteria. Third, we conducted extraction of data in clinical trials using a standardized Excel spreadsheet, when the articles did meet the selection criteria. The accuracy of the extracted data was independently confirmed by a second reviewer. Finally, both the two reviewers determined if the study was to be included in the meta-analysis.

To avoid the inclusion of duplicated data that may lead to an overestimation of treatment effects in final meta-analysis, we carefully appraised and examined the retrieved RCTs by comparison of author names and period of study. Then, we extracted the following characteristics: the name of the first author, year of the publication, diagnostic criteria, types of intervention, treatment duration and age of the participants, number of the participants, and number of the treatment responses in each arm. The information about side effect was also extracted from the relevant articles.

All disagreements were resolved by discussion between the two reviewers. If disagreements continued, they were resolved through seeking the opinion of a third reviewer (H.H.). Where required, the reviewers would try to obtain additional information from the original authors.

### 2.5. Quality Critical Appraisal

Two reviewers (H.G. and F.Q.) conducted a critical appraisal by using the Cochrane risk of bias tool for RCTs, which was recommended by the Cochrane Handbook for Systematic Reviews of Interventions [38]. The Cochrane risk of bias tool for RCTs is a six-item list, which designed to assess (1) sequence generation, (2) allocation concealment, (3) blinding of participants and personnel, (4) blinding of outcome assessment, (5) incomplete data, (6) selective reporting of outcomes, and (7) other potential bias. Each item was rated as criteria met, criteria not met, or unclear whether criteria were met.

### 2.6. Selected Outcomes

Of all the RCTs, the term “patients without symptoms” was defined as having more than 3 bowel movements per week, and stool should be soft and smooth. The term “patients with significant improvement of symptoms” was defined as having 2-3 bowel movements per week, and stool should be soft but poor defecation. And these outcomes were considered successful treatments for patients.

### 2.7. Statistical Analysis

Meta-analysis was performed by using Review Manager (RevMan) version 5.3 (Cochrane Collaboration). Statistical heterogeneity among studies was evaluated with the chi-square and *I*^2^ tests. A fixed-effect model was used when no significant heterogeneity was observed (*P* > 0.1). Additionally, a random-effect model was applied if a significant heterogeneity between individual effect-sizes was found. Otherwise, the data would be synthesized with descriptive statistics rather than quantitative assessment. For dichotomous data, risk ratio (RR) and corresponding 95% confidence intervals (CI) were calculated.

## 3. Results

### 3.1. Literature Search

**Figure 1:**
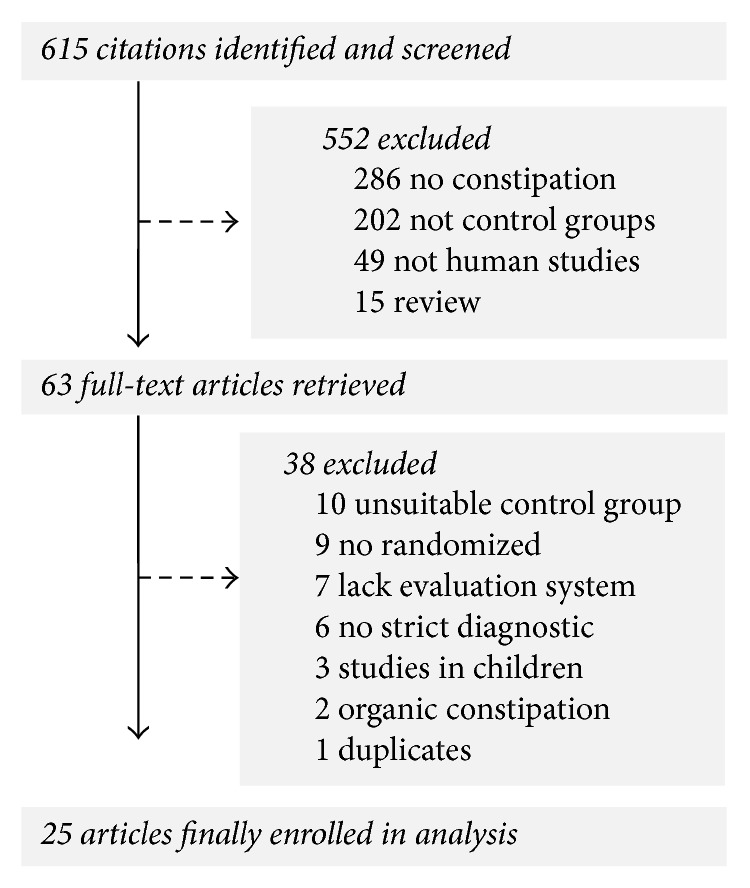
Study selection process for the meta-analysis with specifications of reasons.

An overview of the study selection process is summarized in Figure 1. Literature searches identified 615 potentially relevant abstracts after elimination of duplicates. After review of the abstracts, 63 full-text publications were assessed, of which 25 studies were included in this review, involving a total of 2089 participants. All of these trials took place in China and were reported in Chinese.

### 3.2. Methodological Quality of Studies Included

**Figure 2:**
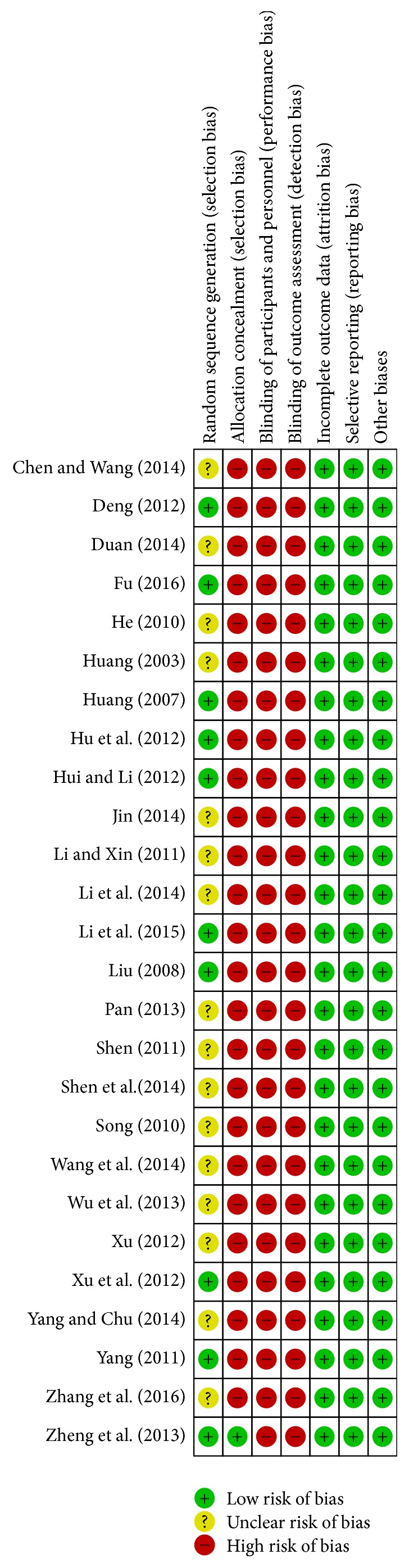
Methodological quality assessment of the risk of bias for each included study.

According to the Cochrane risk of bias assessment tool, the methodologic quality item for all included studies is described in Figure 2. In general, the methodological quality of the 25 studies was low. Of the 25 studies, no randomized, double-blind, and placebo-controlled trial was designed to study. Ten studies used a random number table for randomization, and the other studies did not provide detailed information about the random sequence generation. In addition, all the studies failed to describe the allocation concealment, blinding of participants and personnel, and blinding of outcome assessors in detail. None of the studies reported missing data.

### 3.3. Study Characteristics

Overall, of the 25 studies included in the meta-analysis, 2089 patients had been randomized to either one of the experimental groups (with MBYT) or the control groups (without MBYT). The studies published between 2003 and 2016 were included. In the majority of the studies, patients had chronic constipation. In seven studies, functional constipation was related to underlying disease such as Parkinson's disease [14], chronic obstructive pulmonary disease [30], stroke [25], intertrochanteric fracture of femur [19], irritable bowel syndrome [28], and uterine retroflexion [37].  Table 1 summarizes the design of each of the individual studies. One study was not included in the meta-analysis as the efficacy was showed in the improvement in their symptom score [39]. However, this study was included in the systematic review to evaluate relevant safety.

**Figure 3:**
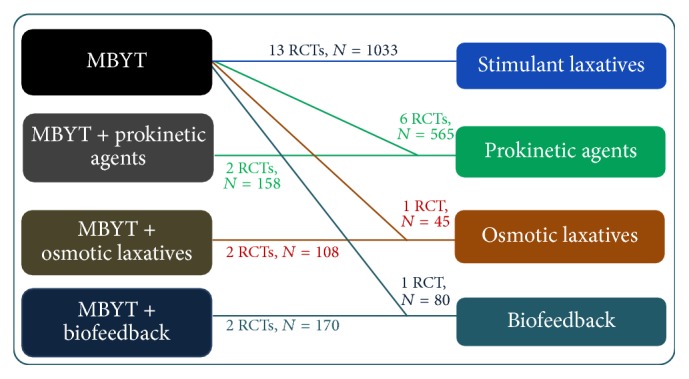
Network formed by interventions and their direct comparisons included in the analyses.

Figure 3 presents the network diagram formed by interventions and their direct comparisons based on the 25 RCTs included in the meta-analysis. There were no placebo only groups in any of the included studies. Of the 25 RCTs, thirteen studies compared MBYT with stimulant laxatives (*N* = 1033); six studies compared MBYT with prokinetic agents (*N* = 565); only one study compared MBYT with osmotic laxatives (*N* = 45); only one study compared MBYT with biofeedback (*N* = 80); only two studies compared MBYT plus prokinetic agents with prokinetic agents (*N* = 158); only two studies compared MBYT plus osmotic laxatives with osmotic laxatives (*N* = 115); only two studies compared MBYT plus biofeedback with biofeedback (*N* = 170). There was one RCT, from which we extracted three groups of data, including MBYT versus prokinetic agents, MBYT versus biofeedback, and MBYT plus biofeedback versus biofeedback [32].

### 3.4. The Efficacy for Functional Constipation

**Figure 4:**
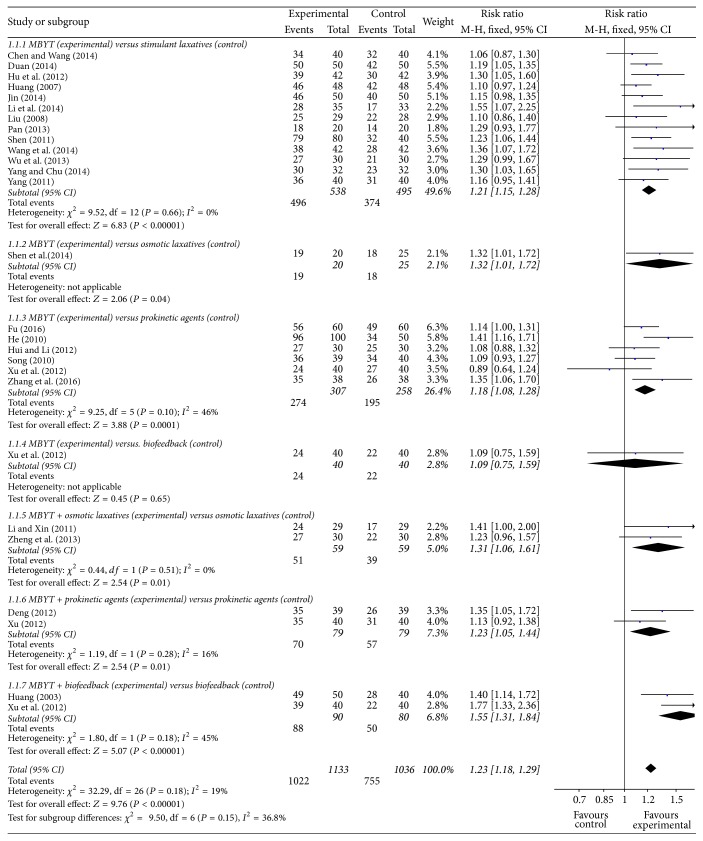
Treatment effects of MBYT on clinical response in patients with functional constipation. Risk ratio > 1.0 indicates that the symptomatic improvement is higher in the experimental group than that in control group. “Events” refers to the number of individuals that received successful treatments. “Total” refers to the total number of individuals. CI, confidence interval; M-H, Mantel-Haenszel method of calculation.

Thirteen randomized controlled trials tested MBYT against stimulant laxatives in patients with functional constipation [14–26]. All trials reported effects in favor of MBYT compared to stimulant laxatives at the end of treatment. As shown in Figure 4, the meta-analysis (*N* = 1033) showed a significant increase of symptom improvement for MBYT compared to stimulant laxatives (RR = 1.21, 95% CI 1.15–1.28; *Z* test = 6.83, *P* < 0.00001). The chi-square test for homogeneity of odds ratios is performed to determine whether there are significant differences among the trials (Chi^2^ = 9.52, df = 12; *P* = 0.66), which indicate that there are no statistically differences in results (Figure 4).

Six RCTs tested MBYT against prokinetic agents in patients with functional constipation [28–33]. A meta-analysis of the trials (*n* = 565) showed a significant increase of symptom improvement compared to prokinetic agents (RR = 1.18, 95% CI 1.08–1.28; *Z* test = 3.88, *P* = 0.0001). As shown in Figure 4, the chi-square test for homogeneity indicates that there are no statistically differences in results among the seven trials (Chi^2^ = 9.25, df = 5; *P* = 0.10).

There is only one study which tested MBYT against osmotic laxatives in patients with functional constipation [27]. The result showed a significant increase of symptom improvement compared to osmotic laxatives (RR = 1.32, 95% CI 1.01–1.72; *Z* test = 2.06, *P* = 0.04).

Xu et al. (2012) trial tested MBYT against biofeedback in patients with functional constipation [32]. The meta-analysis showed no significant increase of symptom improvement compared to biofeedback (RR = 1.09, 95% CI 0.75–1.59; *Z* test = 0.45, *P* = 0.65).

There are only two studies with 59 cases and 59 controls in patients with functional constipation [13, 34]. The results showed a significant increase of symptom improvement for MBYT plus osmotic laxatives, compared to osmotic laxatives (RR = 1.31, 95% CI 1.06–1.61; *Z* test = 2.54, *P* = 0.01). As shown in Figure 4, the chi-square test for homogeneity indicates that there are no statistically differences in results between the two trials (Chi^2^ = 0.44, df = 1; *P* = 0.51).

Two studies tested MBYT plus prokinetic agents against prokinetic agents in patients with functional constipation [35, 36]. A meta-analysis of the trials (*n* = 158) showed a significant increase of symptom improvement for MBYT plus prokinetic agents, compared to prokinetic agents (RR = 1.23, 95% CI 1.05–1.44; *Z* test = 2.54, *P* = 0.01). As shown in Figure 4, the chi-square test for homogeneity indicates that there are no statistically differences in results between the two trials (Chi^2^ = 1.19, df = 1; *P* = 0.28).

Two studies tested MBYT plus biofeedback against biofeedback in patients with functional constipation [32, 37]. A meta-analysis of the trials (*n* = 170) showed a significant increase of symptom improvement for MBYT plus biofeedback, compared to biofeedback (RR = 1.55, 95% CI 1.31–1.84; *Z* test = 5.07, *P* < 0.00001). As shown in Figure 4, the chi-square test for homogeneity indicates that there are no statistically differences in results between the two trials (Chi^2^ = 1.80, df = 1; *P* = 0.18).

### 3.5. Adverse Events

**Figure 5:**
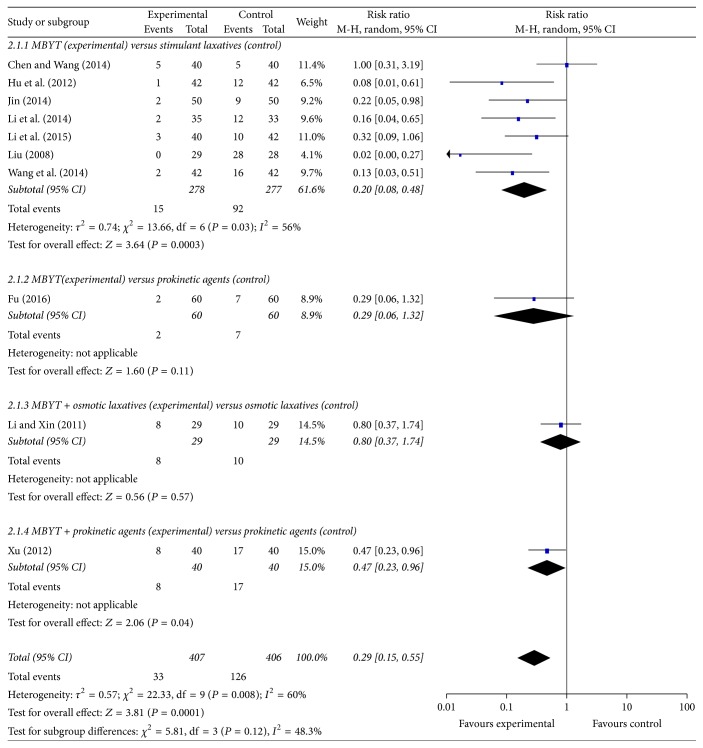
Meta-analysis for MBYT on adverse events in adult patients with functional constipation. Risk ratio < 1.0 indicates that the adverse event is lower in the experimental group than that in control group. The subheading “Events” refers to the number of adverse event. “Total” refers to the total number of individuals.

Of the included trials, ten RCTs reported information on adverse events (Figure 5). Seven RCTs tested MBYT against stimulant laxatives in patients with functional constipation [14, 16, 18–20, 23, 39]. The adverse events were malaise, diarrhea, abdominal pain, drug tolerance, and so on. A random-effect model was used to analyze overall adverse events based on the heterogeneity values (*P* = 0.03, *I*^2^ = 56%). However, the incidence of adverse events was lower in the MBYT group, as compared to the stimulant laxatives group (RR = 0.20, 95% CI 0.08–0.48; *Z* test = 3.64, *P* = 0.0003). As shown in Figure 5, the chi-square test for homogeneity indicates that there are statistically differences in results among the seven trials (Chi^2^ = 13.66, df = 6; *P* = 0.03). Furthermore, the adverse events were also decreased in the MBYT, as compared to prokinetic agents [28]. Interestingly, when MBYT is used as an adjuvant therapy, the adverse events were also decreased in the combination group, when compared with prokinetic agents alone (RR = 0.47, *P* = 0.04) [32], or osmotic laxatives alone [13].

## 4. Discussion

In total, this study assessed the efficacy and safety of MBYT in adult patients with functional constipation. Review Manager 5.3 software was used to analyze the clinical data from 25 randomized controlled trials, with a total of 2089 participants. All trials were carried out in China, and all the patients involved were Chinese. The current data indicated that MBYT had excellent therapeutic effect in adult patients with functional constipation, when compared with stimulant laxatives, osmotic laxatives, and prokinetic agents. The data also demonstrated that, when used as an adjuvant therapy, MBYT could significantly improve the symptoms of functional constipation. Furthermore, the study suggests that functional constipation patients taking MBYT experienced fewer adverse events.

Functional constipation is a very common condition, and it has a negative effect on patients' quality of life. The condition is a huge health care burden, with a significant impairment of both mental and physical components. The pathophysiology of functional constipation is poorly understood and likely multifactorial. The management of functional constipation remains challenging for both clinician and patients. Recent studies have demonstrated that bulk (fiber) laxatives, osmotic laxatives, stimulant laxatives, stool softeners, prokinetic agents, lifestyle changes, and biofeedback therapy are commonly used for the management of functional constipation [3, 40]. Exercise and dietary fiber are helpful in some patients with functional constipation [41]. Laxatives including bulking agents, stool softeners, osmotic agents, and stimulant laxatives have been found to be more effective than placebo at relieving symptoms of functional constipation [42]. Prokinetic agents can reduce the need of laxatives and show a tendency to normalize stool consistency [43]. However, a substantial number of patients (up to 47%) are not completely satisfied with these treatments. The main reasons are treatment efficacy, inconsistent symptom response, and concerns with regard to safety, adverse effects, taste, inconvenience, and cost [44]. Therefore, a new agent that possibly works through other pathways could be helpful for patients unable to tolerate these therapies.

MBYT is a classic herbal formula that has been commonly prescribed for patients with functional constipation in Eastern Asian countries for approximately 800 years. To our knowledge, this was the first systematic review to critically evaluate the efficacy and safety of MBYT for functional constipation. Indeed, as shown in Figure 4, the present study showed that MBYT had produced positive results in functional constipation. Analyses of subgroups revealed that the effects of MBYT monotherapy were superior to stimulant laxatives (RR = 1.21), osmotic laxatives (RR = 1.32), and prokinetic agents (RR = 1.18). There was also some evidence that the effects of combination MBYT therapy were considered to be superior to osmotic laxatives alone (RR = 1.31), and prokinetic agents alone (RR = 1.23). Moreover, biofeedback therapy is a well-known and effective therapeutic treatment for functional constipation [45]. When evaluating the efficacy of MBYT plus biofeedback therapy, patients with MBYT display over 1.55-fold higher probability of symptom relief, as compared to patients with biofeedback treatment alone. In addition, patients taking MBYT experienced fewer adverse events of malaise, diarrhea, abdominal pain, and drug tolerance compared to the control groups.

The present study has several potential limitations that should be addressed. First, all the RCTs in the present study came from mainland China and were written in Chinese, which were therefore not accessible by the international research community. Second, there is no high quality RCTs in the present study; all included studies were of low to moderate quality (Figure 2). As mentioned in the previous meta-analyses for herbal formula [46], many RCTs did not provide detailed demographic and methodological information (e.g., durations of illness, medication history, sequence generation, and allocation concealment). Third, quality control of herbal formula has been necessary and urgent for its application and development and is very important to ensure its safety and efficacy, but all the RCTs lacked sufficient information on the quality control of MBYT. Fourth, according to the theory of traditional Chinese medicine, the clinical herbalist can combine other herbs based on a diagnosis, to fit each individual's complaint and constitution. In addition to the eight herbs mentioned above, it also contains other herbs, which will affect the overall efficacy evaluation of MBYT. Finally, it needs to be further studied in double-blind trials and placebo-controlled trials to exclude psychological effects, which may play a very important role in the treatment of functional constipation.

## 5. Conclusion

The meta-analysis provides strong evidence that herbal formula MBYT appears to have excellent therapeutic effect on functional constipation, and no serious side effects were identified. Our data suggest that MBYT could be considered an effective and safe alternative treatment for adult patients with functional constipation. However, due to overall limited quality of the included studies, the therapeutic benefit of MBYT can be substantiated to a limited degree. Clinical trials with better methodological quality, larger sample size, and longer follow-up periods are recommended in further research for MBYT.

## Figures and Tables

**Table 1:** Characteristics of the included studies in meta-analysis.

Study	Diagnostic criteria	Type of the control group	Treatment duration	Age (years)		Percentage of men		Intervention drugs		Symptom improvement		Adverse event
				Case	Control	Case	Control	Case	Control	Case	Control	
Chen and Wang, 2014 [14]	DESS	Stimulant laxatives	4 weeks	55–78	53–79	55	48	MBYT (36.0 g, DE, TID)	Phenolphthalein (200 mg, TA, QD)	85.0% (34/40)	80.0% (32/40)	Included
Duan, 2014 [15]	CGND/RIIIC	Stimulant laxatives	3 weeks	57–69	56–68	50	46	MBYT (45.0 g, DE, TID)	Phenolphthalein (100 mg, TA, QD)	100.0% (50/50)	84.0% (42/50)	NR
Hu et al., 2012 [16]	CGND	Stimulant laxatives	4 weeks	68.1 ± 10.6	66.6 ± 11.1	40	36	MBYT (38.0 g, DE, BID)	Phenolphthalein (200 mg, TA, QD)	92.9% (39/42)	71.4% (30/42)	Included
Huang, 2007 [17]	DESS	Stimulant laxatives	2 weeks	42.0 ± 1.0	42.6 ± 1.2	46	44	MBYT (69.5 g, DE, BID)	Phenolphthalein (200 mg, TA, QD)	95.8% (46/48)	87.5% (42/48)	NR
Jin, 2014 [18]	DESS	Stimulant laxatives	4 weeks	59–84	59–83	66	64	MBYT (41.0 g, DE, BID)	Phenolphthalein (200 mg, TA, QD)	92.0% (46/50)	80.0% (40/50)	Included
Li et al., 2014 [19]	DESS	Stimulant laxatives	3 weeks	66–82	65–84	54	70	MBYT (28.5 g, DE, BID)	Phenolphthalein (200 mg, TA, QD)	80.0% (28/35)	51.5% (17/33)	Included
Liu, 2008 [20]	DESS	Stimulant laxatives	4 weeks	55–82	52–80	38	32	MBYT (60.5 g, DE, BID)	Phenolphthalein (200 mg, TA, QD)	86.2% (25/29)	78.6% (22/28)	Included
Pan, 2013 [21]	DESS/RIIC	Stimulant laxatives	2 weeks	62–76	63–75	60	55	MBYT (56.5 g, DE, BID)	Phenolphthalein (100 mg, TA, QD)	90.0% (18/20)	70.0% (14/20)	NR
Shen, 2011 [22]	DESS	Stimulant laxatives	10 days	60–78	60–80	75	70	MBYT (40.0 g, DE, BID)	Bisacodyl (5 mg, TA, TID)	98.8% (79/80)	80.0% (32/40)	NR
Wang et al., 2014 [23]	DESS	Stimulant laxatives	1 week	57–87	54–86	71	64	MBYT (38.0 g, DE, BID)	Phenolphthalein (200 mg, TA, QD)	90.5% (38/42)	66.7% (28/42)	Included
Wu et al., 2013 [24]	CGND/RIIIC	Stimulant laxatives	2 weeks	50.3 ± 19.2	48.4 ± 25.3	37	50	MBYT (64.5 g, DE, BID)	Phenolphthalein (100 mg, TA, QD)	90.0% (27/30)	70.0% (21/30)	NR
Yang and Chu 2014 [25]	CGND	Stimulant laxatives	5 weeks	45–81	42–83	53	50	MBYT (60.5 g, DE, BID)	Bisacodyl (5 mg, TA, QD)	93.8% (30/32)	71.9% (23/32)	NR
Yang, 2011 [26]	DESS	Stimulant laxatives	2 weeks	65.2 ± 2.3	63.9 ± 2.0	45	42	MBYT (48.5 g, DE, BID)	Phenolphthalein (200 mg, TA, QD)	90.0% (36/40)	77.5% (31/40)	NR
Shen et al., 2014 [27]	CGND	Osmotic laxatives	1 week	22–71	22–71	58	58	MBYT (47.5 g, DE, BID)	Duphalac (10 g, SO, BID)	95.0% (19/20)	72.0% (18/25)	NR
Fu, 2016 [28]	CGND/DESS	Prokinetic agents	4 weeks	32.5 ± 10.1	31.6 ± 9.8	58	57	MBYT (41.0 g, DE, BID)	Cisapride (5 mg, TA, TID)	93.3% (56/60)	81.7% (49/60)	Included
He, 2010 [29]	DESS	Prokinetic agents	2 weeks	18–72	18–72	42	42	MBYT (64.5 g, DE, BID)	Cisapride (5 mg, TA, BID)	96.0% (96/100)	68.0% (34/50)	
Hui and Li, 2012 [30]	DESS	Prokinetic agents	2 weeks	65.4 ± 6.3	67.3 ± 5.8	63	67	MBYT (47.5 g, DE, BID)	Cisapride (5 mg, TA, TID)	90.0% (27/30)	83.3% (25/30)	NR
Song, 2010 [31]	CGND/DESS /RIIC	Prokinetic agents	4 weeks	43.6 ± 5.1	45.1 ± 5.3	56	55	MBYT (70.0 g, DE, BID)	Cisapride (5 mg, TA, TID)	92.3% (36/39)	85.0% (34/40)	NR
Xu et al., 2012 [32]	DESS/RIIIC	Prokinetic agents	20 days	80.9 ± 6.4	79.6 ± 7.9	50	50	MBYT (3.0 g, PI, TID)	Mosapride (10 mg, TA, TID)	60.0% (24/40)	67.5% (27/40)	NR
		Biofeedback	20 days	75.7 ± 9.7	79.2 ± 7.7	48	48	MBYT (3.0 g, PI, TID) + Biofeedback (QD)	Biofeedback (QD)	97.5% (39/40)	55.0% (22/40)	NR
Zhang et al., 2016 [33]	CGND/DESS /RIIIC	Prokinetic agents	60 days	18–66	18–68	53	47	MBYT (28.5 g, DE, BID)	Cisapride (5 mg, TA, TID)	92.1% (35/38)	68.4% (26/38)	NR
Li and Xin, 2011 [13]	DESS/RIIIC	Osmotic laxatives	4 weeks	45–86	45–86	48	48	MBYT (3.0 g, GR, BID) + Duphalac (667 mg, SO, BID)	Duphalac (667 mg, SO, BID)	28.8% (24/29)	58.6% (17/29)	Included
Zheng et al., 2013 [34]	CGND/DESS /RIIIC	Osmotic laxatives	3 weeks	30–80	29–77	57	53	MBYT (120.0 g, DE, QD) + PEG4000 (20 g, PO, QD)	PEG4000 (20 g, PO, QD)	90.0% (27/30)	73.3% (22/30)	Included
Deng, 2012 [35]	CGND	Prokinetic agents	3 weeks	59–78	57–79	41	46	MBYT (50.5 g, DE, BID) + Cisapride (10 mg, TID)	Cisapride (10 mg, TA, TID)	89.7% (35/39)	66.7% (26/39)	NR
Xu, 2012 [36]	DESS/RIIIC	Prokinetic agents	4 weeks	>60	>60	60	55	MBYT (34.3 g, DE, TID) + Mosapride (5 mg, TA, TID)	Mosapride (5 mg, TA, TID)	87.5% (35/40)	77.5% (31/40)	Included
Huang, 2003 [37]	DESS	Biofeedback	2 weeks	35–50	35–50	0	0	MBYT (3.0 g, PI, QD) + Kegel exercises (QD)	Kegel exercises (QD)	98.0% (49/50)	70.0% (28/40)	NR

MBYT: Modified Buzhong-Yiqi-Tang; CGND: clinical guideline of new drugs for TCM; DESS: diagnostic efficacy of standard TCM syndrome; RIIC: Rome II Criteria; RIIIC: Rome III Criteria; DE, decoction; GR, granules; PI, pill; PO, powder; SO, solution; TA, tablet; QD, once a day; BID, twice a day; TID, three times a day; NR, no record.
